# Long-term treatment of hereditary transthyretin amyloidosis with patisiran: multicentre, real-world experience in Italy

**DOI:** 10.1007/s10072-024-07494-9

**Published:** 2024-04-16

**Authors:** Luca Gentile, Anna Mazzeo, Chiara Briani, Silvia Casagrande, Marcella De Luca, Gian Maria Fabrizi, Christian Gagliardi, Chiara Gemelli, Francesca Forcina, Marina Grandis, Valeria Guglielmino, Giacomo Iabichella, Luca Leonardi, Alessandro Lozza, Fiore Manganelli, Roberta Mussinelli, Filomena My, Giuseppe Occhipinti, Silvia Fenu, Massimo Russo, Angela Romano, Alessandro Salvalaggio, Matteo Tagliapietra, Stefano Tozza, Giovanni Palladini, Laura Obici, Marco Luigetti

**Affiliations:** 1 Unit of Neurology and Neuromuscular Diseases, Department of Clinical and Experimental Medicine, University of Messina, Messina, Italy; 2 Department of Neurosciences, Neurology Unit, University of Padova, Padua, Italy; 3 Department of Neurosciences, Psychology, Drug Research and Child Health (NEUROFARBA), University of Florence, Florence, Italy; 4 Department of Neurological Sciences, Biomedicine and Movement Sciences, University of Verona, Verona, Italy; 5 Cardiology Unit, IRCCS Azienda Ospedaliero-Universitaria of Bologna, Bologna, Italy; 6 IRCCS Policlinico San Martino Hospital, Genoa, Italy; 7 Department of Neuroscience, Mental Health and Sensory Organs (NESMOS), Sapienza University of Rome, Rome, Italy; 8 Dipartimento Di Neuroscienze, Riabilitazione, Oftalmologia, Genetica e Scienze Materno-Infantili, Università Di Genova, Genoa, Italy; 9 Dipartimento Di Neuroscienze, Università Cattolica del Sacro Cuore, Rome, Italy; 10 Amyloidosis Research and Treatment Centre, IRCCS Fondazione Policlinico San Matteo, Pavia, Italy; 11 Department of Neuroscience, Reproductive and Odontostomatological Science, University of Naples “Federico II”, Naples, Italy; 12 Department of Neurology, “Vito Fazzi” Hospital, Lecce, Italy; 13 S.C. Malattie Neurologiche Rare, Dipartimento di Neuroscienze Cliniche, Fondazione IRCCS Istituto Neurologico Carlo Besta, Milan, Italy; 14 Dipartimento Di Neuroscienze, Organi Di Senso e Torace, Fondazione Policlinico Universitario Agostino Gemelli IRCCS, Rome, Italy; 15 Department of Molecular Medicine, University of Pavia, Pavia, Italy

**Keywords:** Hereditary transthyretin amyloidosis, Real-world, Compassionate use programme, Patisiran

## Abstract

**Background:**

Hereditary transthyretin (ATTRv, v for variant) amyloidosis with polyneuropathy is a rare disease caused by mutations in the transthyretin gene. In ATTRv amyloidosis, multisystem extracellular deposits of amyloid cause tissue and organ dysfunction. Patisiran is a small interfering RNA molecule drug that reduces circulating levels of mutant and wild-type TTR proteins. Prior to its regulatory approval, patisiran was available in Italy through a compassionate use programme (CUP). The aim of this study was to analyse the long-term outcomes of patients who entered into the CUP.

**Methods:**

This was a multicentre, observational, retrospective study of patients with ATTRv amyloidosis treated with patisiran. The analysis included change from baseline to 12, 24, 36 and 48 months in familial amyloid polyneuropathy (FAP) stage, polyneuropathy disability (PND) class, neuropathy impairment score (NIS), modified body mass index (mBMI), Compound Autonomic Dysfunction Test (CADT), Karnofsky Performance Status (KPS) scale and Norfolk Quality of Life–Diabetic Neuropathy (QoL-DN) questionnaire. Safety data were also analysed.

**Results:**

Forty patients from 11 Italian centres were enrolled: 23 in FAP 1 (6 in PND 1 and 17 in PND 2) and 17 in FAP 2 (8 in PND 3a and 9 in PND 3b) stage. In this population, the mean NIS at baseline was 71.4 (± 27.8); mBMI, 917.1 (± 207) kg/m^2^; KPS, 67.1 (± 14.0); Norfolk QoL-DN, 62.2 (± 25.2); and CADT, 13.2 (± 3.3). Statistical analysis showed few significant differences from baseline denoting disease stability. No new safety signals emerged.

**Conclusions:**

Patisiran largely stabilised disease in patients with ATTRv amyloidosis.

**Supplementary Information:**

The online version contains supplementary material available at 10.1007/s10072-024-07494-9.

## Introduction

Hereditary transthyretin (ATTRv, v for variant) amyloidosis is a rare, progressive and ultimately lethal autosomal dominant disorder [1]. Transthyretin (TTR) circulates as a tetrameric protein produced by the liver. Mutations in the *TTR* gene destabilise the native structure of the protein, causing its misfolding with a consequent dissociation of the tetramer and accumulation of amyloid deposits containing mutated and wild-type TTR protein in the extracellular space [1, 2]. The most common *TTR* variant involves valine 30 which is replaced by a methionine (V30M). About 50% of patients with ATTRv amyloidosis carry this mutation, which has been linked to both an early (30–55 years of age) and a late onset of disease [3, 4]. Amyloid deposits as well as toxic TTR monomers and oligomers cause extensive tissue damage, mostly leading to polyneuropathy and cardiomyopathy [5–8]. Autonomic neuropathy can also be part of the clinical picture. Autonomic nerves can be affected early in the disease course, i.e., prior to the motor nerves, and the presence of dysautonomia can be used in differential diagnosis of progressive neurological conditions [9]. Other organs possibly involved are the kidney and gastrointestinal tract [10–13].

Current treatments in ATTRv amyloidosis include disease-modifying drugs such as TTR stabilisers and *TTR* gene-silencing drugs, namely patisiran, vutrisiran and inotersen. The only TTR stabiliser approved for ATTRv amyloidosis is tafamidis, with the indication for the treatment of patients with stage 1 polyneuropathy at the dose of 20 mg [14]. Tafamidis is also available for the treatment of cardiomyopathy associated with ATTR amyloidosis at the dose of 61 mg, both in hereditary and wild-type disease [14]. Conversely, at present, three TTR gene silencers are approved for patients with ATTRv amyloidosis with neurological or mixed phenotype and stage 1 or 2 polyneuropathy [15].

Patisiran is a small interfering RNA (siRNA) molecule enclosed in a lipid nanoparticle. The siRNA molecules are small double-stranded RNAs that exert their effect by separating into single strands and binding to target messenger RNA (mRNA) sequences, ultimately causing target mRNA to break and degrade, further halting translation and inducing gene suppression by the short RNA strands [16]. Patisiran is administered to patients as a slow IV infusion at the dose of 0.3 mg/kg of body weight every 3 weeks. The efficacy of patisiran in ATTRv amyloidosis has been confirmed in numerous studies [17–22]. The clinical response may occur already after 6 months from treatment commencement [23].

EMA approved the use of patisiran in 2018. Initial access to the drug in Italy was available through an early access and compassionate use programmes (CUP). The aim of this study was to collect and analyse data deriving from the clinical evaluation of patients affected by ATTRv amyloidosis and treated with patisiran for up to 48 months.

## Materials and methods

### Study design

The study was a multicentric, retrospective, observational study of patients with ATTRv amyloidosis treated with patisiran within the CUP who continued the treatment with patisiran after its regulatory approval. The analysis includes patients who received patisiran treatment between May 2018 and December 2023.

### Patients

The study took place in 11 Italian centres (see Supplementary Table S1 for the full list of centres involved). Patients with ATTRv amyloidosis treated with patisiran within CUP were enrolled into the study. Patients had to provide an informed consent to take part in the study. The exclusion criteria included familial amyloid polyneuropathy (FAP) stage 3 disease, nonadherence due to complications independent of ATTRv amyloidosis and missing follow-up. Patients underwent routine clinical neurological evaluations. All parameters were evaluated at baseline and at 12, 24, 36 and 48 months of follow-up. Data collection at 60 months was also attempted but found to be immature for the majority of patients.

### Study endpoints

Efficacy endpoints were the change in FAP stage, polyneuropathy disability (PND) class, Neuropathy Impairment Score (NIS), modified body mass index (mBMI), Compound Autonomic Dysfunction Test (CADT), Karnofsky Performance Status (KPS) scale and Norfolk Quality of Life–diabetic neuropathy (QoL-DN) questionnaire from baseline to 12, 24, 36 and 48 months.

FAP stages derive from a study by Coutinho et al. who classified three disease stages based on walking capacity: FAP 1 describes a symptomatic, but fully ambulatory patient, FAP 2 stage is defined by the need for walking aids, and FAP 3 stage denotes a wheelchair dependence or being bedridden [24]. PND class is used to describe ambulatory dysfunction due to neurological impairment. Patients are assigned a score of 1, 2, 3a, 3b or 4, ranging from no problems with walking (PND 1) to being wheelchair-bound or bedridden (PND 4) [25]. NIS allows for evaluation of the neurologic impairment in patients with ATTRv amyloidosis based on the assessment of muscle strength, reflexes and sensation in the limbs; higher scores indicate greater impairment on a 0 to 244 scale [26]. CADT was developed to evaluate the symptoms of autonomic dysfunction encountered in patients with ATTRv amyloidosis; low scores correspond to severe dysfunction [27]. KPS is a measure of a functional impairment. It is scored in increments of 10% from 0 (death) to 100% (no disease) [28]. Norfolk QoL-DN is used to assess neuropathy-related QoL and is composed of a part dedicated to symptoms and another part dedicated to the impact of symptoms on daily living. High scores denote poor QoL [29].

### Statistical analysis

Statistical analysis was performed using SPSS version 21.0 statistical package. Data are presented as means with standard deviations (SD), medians with interquartile ranges (IQR) or percentages, as appropriate. A classical inferential approach (paired *t*-test) was used for the pair baseline and each of the three timepoints. For nonnormally distributed variables, the Wilcoxon signed-ranked test was used as a nonparametric significance test. A *p*-value of < 0.05 was considered statistically significant.

## Results

### Baseline characteristics

**Fig. 1:**
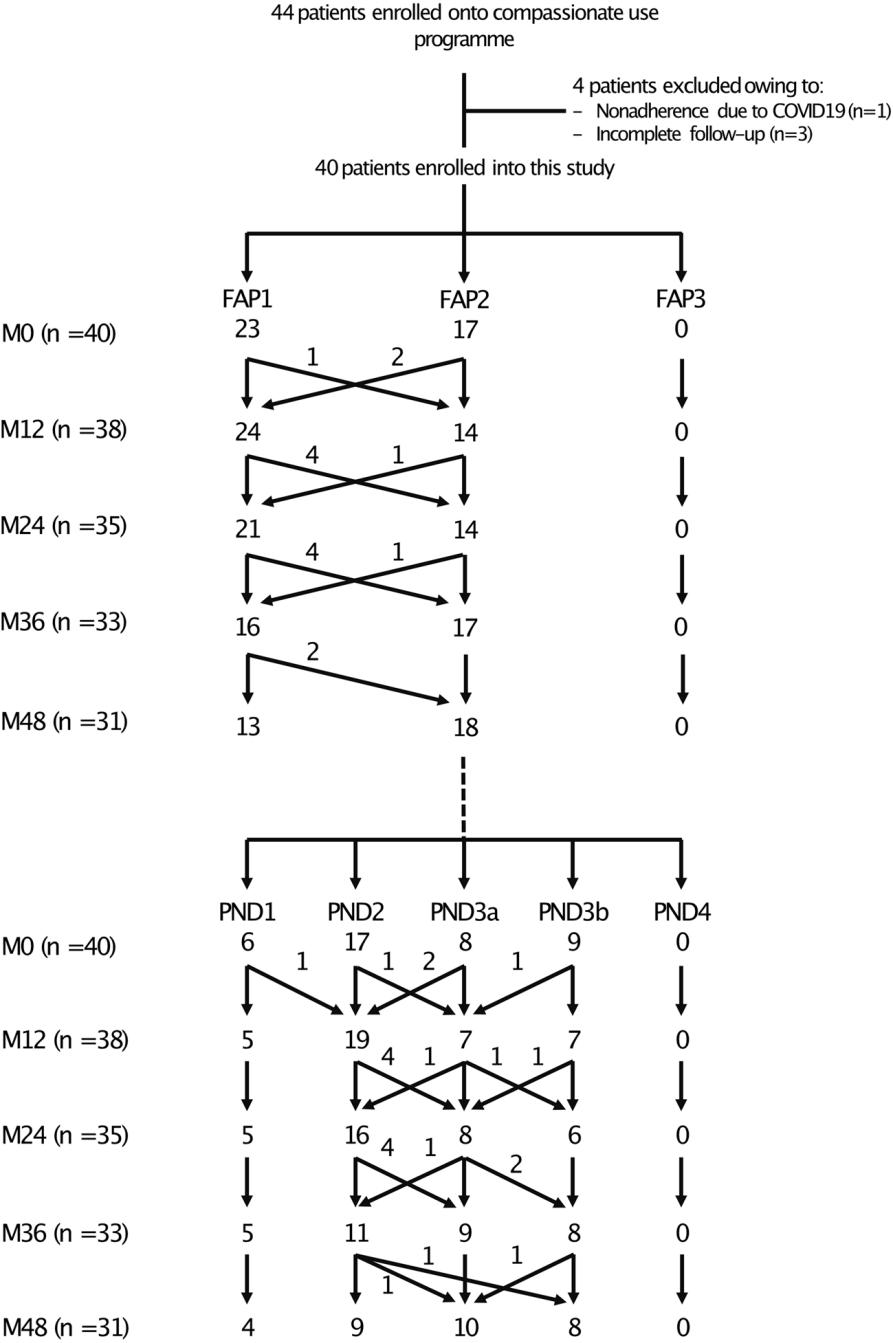
Patients’ enrolment and the evolution of the familial amyloid polyneuropathy (FAP) stage and polyneuropathy disability (PND) score during the follow-up. M, month

Forty-four patients received patisiran within CUP and were enrolled into the study. Four were excluded from the analysis due to incomplete follow-up or COVID-19 complications resulting in nonadherence (Fig. 1 the two parts of the figure (FAP and PND) are repeated twice). Demographics, disease duration at the time of entering CUP, the exact *TTR* gene mutation and baseline characteristics pertaining to disease status are given in Table 1 and in Supplementary Table S2. Thirty-four (85%) patients were taking tetramer stabilisers at the time of initiating patisiran, but none of them received double therapy once started on patisiran. In fact, patients were switched to patisiran because of disease progression on tafamidis. At the onset of the study, 23 and 17 patients had FAP 1 and FAP 2 stage disease, respectively, whereas the distribution of the PND scores was 6 (15%), 17 (42.5%), 8 (20%) and 9 (22.5%) patients with scores 1, 2, 3a or 3b, respectively (Fig. 1). Cardiac involvement was demonstrated in 70% of patients (Supplementary Table S2).

**Table 1:** Baseline characteristics of the study population and disease status

Parameter	Number of patients (%)
Age (years)	
Median (IQR)	70 (65.5; 75.9)
Gender	
Female	10 (25%)
Male	30 (75%)
*TTR* genotype	
V30M	10 (25%)
Non V30M	30 (75%)
F64L	17 (42.5%)
E89Q	6 (15%)
Y78F	2 (5%)
Others*	5 (12.5%)
Disease onset before the age of 50	
Yes	7 (15.9%)
No	37 (84.1%)
Time from diagnosis to entering CUP (months)	
Median (IQR)	48 (25; 71)
Cardiomyopathy at diagnosis	
Yes	28 (70%)
No	12 (30%)
Use of tetramer stabilisers at the time of initiating the treatment with patisiran	
Yes	34 (85.0%)
No	6 (15.0%)**

*CUP*, compassionate use programme; *IQR*, interquartile range

*Other mutations included: T49A, F64I, E89K, S77Y, V122I; each present in one patient

**Tetramer stabilisers previously suspended

### Effectiveness

The details of the evolution of the FAP stage and PND class in the patient population are shown in Fig. 1. Supplementary Table S3 shows the evolution of the two scores in individual patients. At 48 months of follow-up, FAP stage and PND class were available in 31 patients. FAP stage was stable in 25 (80.6%) patients; PND class was stable in 22/31 (70.9%) and improved in one (Supplementary Figure S1 and Supplementary Table S3).

In this population, the mean NIS score at baseline was 71.4 (± 27.8); mBMI, 917.1 (± 207); KPS score, 67.1 (± 14.0); Norfolk QoL-DN questionnaire score, 62.2 (± 25.2); and CADT score, 13.2 (± 3.3). Table 2 shows the mean and SD values of all parameters at 12, 24, 36 and 48 months. Statistical analysis showed few significant differences from baseline to the four timepoints for the parameters analysed (Table 2). These included differences from baseline in NIS and KPS at 36 and 48 months, and Norfolk QoL-DN and CADT at 12 and 24 months. Supplementary Figures S2-S6 show the evolution of scores at all timepoints for all the parameters in individual patients. At the end of the follow-up of 48 months, most of patients showed improved or stable mBMI, KPS, Norfolk QoL-DN and CADT scores (Table 3, Supplementary Figs. 2B and 6B). The 60-month follow-up data were mature in only 14 patients and, therefore, were not analysed.

**Table 2:** Study endpoints

Parameter	Baseline	Month 12	Month 24	Month 36	Month 48
NIS (mean (± SD))	71.4 (± 27.7) *N* = 40	68.2 (± 28.6) *N* = 36	70.4 (± 28.7) *N* = 35	76.3 (± 31.1)* *N* = 33	77.5 (± 28.9)* *N* = 31
mBMI (kg/m^2^) (mean (± SD))	917.1 (± 207.1) *N* = 40	934.1 (± 191.7) *N* = 36	925.6 (± 163.1) *N* = 34	961.0 (± 194.6) *N* = 28	941.8 (± 178.1) *N* = 24
KPS (mean (± SD))	67.1 (± 14.0) *N* = 40	63.0 (± 22.1) *N* = 37	65.9 (± 13.0) *N* = 34	63.6 (± 13.6)*^,#^ *N* = 33	62.6 (± 14.1)*^,#^ *N* = 31
Norfolk QoL-DN (mean (± SD))	62.2 (± 25.2) *N* = 37	57.6 (± 25.2)*^,##^ *N* = 34	56.8 (± 23.8)**^,##^ *N* = 33	62.0 (± 24.4) *N* = 31	63.3 (± 25.2) *N* = 29
CADT (mean (± SD))	13.2 (± 3.3) *N* = 39	13.8 (± 3.2)^#^ *N* = 36	14.0 (± 3.4)^#^ *N* = 34	13.6 (± 3.0) *N* = 31	12.7 (± 3.9) *N* = 29

*mBMI*, modified body mass index; *KPS*, Karnofsky performance status; *PND*, polyneuropathy disability; *NIS*, neuropathy impairment score; *CADT*, Composite Autonomic Dysfunction Test; *SD*, standard deviation; *QoL-DN*, Quality Of Life–Diabetic Neuropathy

*p< 0.05, **p < 0.01 in paired *t*-test versus baseline

^#^*p* < 0.05, ^##^*p* < 0.01 in the Wilcoxon signed-rank test versus baseline

**Table 3:** Number and percentage of patients who improved, remained stable or worsened with respect to baseline at the end of follow-up

Parameter	Change from baseline at 48 months
NIS	
< baseline − 2 points	6 (19.4%)
= baseline ± 2 points	6 (19.4%)
> baseline + 2 points	19 (61.3%)
mBMI (kg/m^2^)	
< baseline − 10%	1 (4.2%)
= baseline ± 10%	21 (87.5%)
> baseline + 10%	2 (8.3%)
KPS	
< baseline	13 (41.9%)
= baseline	16 (51.6%)
> baseline	2 (6.5%)
Norfolk QoL-DN	
< baseline	15 (55.6%)
= baseline	1 (3.7%)
> baseline	11 (40.7%)
CADT	
< baseline − 1 point	8 (27.6%)
= baseline ± 1 point	13 (44.8%)
> baseline + 1 point	8 (27.6%)

*mBMI*, modified body mass index; *KPS*, Karnofsky performance status; *PND*, polyneuropathy disability; *NIS*, neuropathy impairment score; *CADT*, Composite Autonomic Dysfunction Test; *QoL-DN*, Quality Of Life–Diabetic Neuropathy

### Safety

A total of 9/40 (22.5%) patients experienced treatment-emergent adverse events when treated with patisiran. The majority of the adverse events encountered were serious, severe in intensity and unrelated to the study drug. Amongst the serious adverse events, two patients with cardiomyopathy at diagnosis experienced a stroke, which was fatal in one case. In one patient, stroke was cryptogenic (no evidence of atrial fibrillation on loop recorder); the patient was treated with acetylsalicylic acid (ASA) and recovered. The other patient experienced atrial fibrillation leading to stroke but was not on oral anticoagulation or ASA prophylaxis due to a past episode of gastric bleeding. Four patients died during the study. Death was considered unrelated to the study drug in one case and unlikely related in the other three patients. Patisiran-related adverse events were mild in intensity and included injection site reactions (extravasation in one case and panniculitis due to extravasation in another).

In four patients, patisiran was discontinued due to adverse events (sepsis, stroke and pneumonia, recurrent vomiting, severe diarrhoea leading to hypovolemic shock). The administration was resumed in one patient in whom the adverse event was resolved. The adverse events and their relatedness to the study drug are shown in Supplementary Table S4.

## Discussion

This is a report of 40 patients with ATTRv amyloidosis treated with patisiran within CUP. Effectiveness analysis showed a substantial stability of NIS, mBMI, KPS, Norfolk and CADT scores during a 4-year treatment period. Of the 31 patients with 48-month follow-up data available, the FAP stage was stable in 80.6% of patients; four (12.9%) patients transiently improved at some point of the follow-up but ultimately returned to their baseline stage. The PND class was stable in 70.9% of patients, and similarly, four (12.9%) patients transiently improved, one of whom from PND 3b at baseline, to PND 3a at 12 months and PND 2 at 24 months. None of the patients described entered the FAP 3 stage or the PND 4 class.

These data are not very different from those obtained in the APOLLO trial, in which at 18 months 65% of patients had a stable PND class, 8% improved their score and 27% worsened [17]. In the long-term open-label (OLE) study at 36 months of follow-up, most patients remained ambulatory (PND < 4), and 55.5% of the patients from the APOLLO-patisiran and 80.0% of those from the phase 2 OLE groups had stabilised or improved ambulation than in the APOLLO-placebo group (42.9%). [22]. At 60 months of follow-up in the OLE study, PND class improved in 4.8% of patients in the APOLLO-placebo and in 9.6% of patients in the APOLLO-patisiran groups and in 13.6% of those in the phase 2 OLE group and worsened in 38.1%, 37.2% and 22.7%, respectively, as reported in the online ClinTrials.gov database [30].

The difference between baseline and 48-month timepoint in NIS score in our cohort reached a statistical significance: such a result was caused by the presence of two cases who substantially worsened over the course of follow-up. One of our patients, who presented at the age of 58 and worsened by 43 points over 48 months of follow-up, had a rare genotype (E89K) with a heavy autonomous nervous system impairment and ocular involvement. This variant, which is endemic in Spain, was found to be associated with near complete penetrance with an onset by the age of 60 years and mixed cardiac and neurologic phenotype with frequent ocular involvement. Patients with E89K mutation tend to have a poor prognosis, if not treated with disease-modifying therapies [31]. Another patient, a male with V30M mutation, with severe disease at baseline (PND 3b; NIS 88.5) experienced a substantial worsening (NIS 112.5; change from baseline of 24 points) at the last follow-up.

A study of the natural history of ATTRv amyloidosis showed that the disease may have a rapid progression with an estimated rate of NIS worsening of 14.3 points/year in a population with a median NIS of 32 [4]. The patient population described in the present study changed from a mean NIS score of 71.4 (± 27.7) to 77.5 (± 28.9), i.e., for a mean change of 4.7 (± 12.5) over a period of 4 years, implying a substantial stabilisation of the disease. These data are similar to those obtained in another real-world treatment experience in a single-centre cohort of patients who received treatment with tafamidis and were later switched to patisiran. Whilst NIS increased by an average of 24.87 (± 17.08) points after 12 months over the tafamidis treatment period, upon the switch to patisiran, NIS increased at a slower rate of an average of 2.4 (± 12.8) points after 12 months, resulting in the stabilisation of neurologic impairment in two-thirds of patients (stable walking status since last evaluation) [32]. The NIS stability data presented by us are better than the change from baseline to 60 months reported in the OLE study, in which it equalled 11.45 (± 16.6) points for patients in the APOLLO-placebo, 10.72 (± 13.6) points for patients in the APOLLO-patisiran groups and 11.18 (± 17.5) points for those in the phase 2 OLE group [30]. In this study, 18 patients have not yet reached the 60-month follow-up, and thus, the 60-month follow-up data are not reported.

Our data on polyneuropathy stabilisation are comparable with those observed in a population with a similar baseline NIS range [17]. Moreover, we also observed an improvement of neurological status in some patients; NIS improved by at least 2 points from baseline in 19.4% of patients, which is less than the finding of improvements in mNIS + 7 in 56% of patients in the APOLLO study at 18 months (Supplementary Figure S2) [17]. It is, however, important to underline that in our study, the observation period was considerably longer, i.e., 48 versus 18 months.

The APOLLO study showed that patients with higher baseline NIS values continued to exhibit more severe neuropathy than patients with lower NIS baseline values when treated with patisiran for 18 months. In fact, patients with lower NIS quartiles treated with patisiran earlier in the disease course maintained better modified NIS + 7, NIS total score, Rasch-built overall disability scale, 10-m walk test, grip strength and Norfolk QOL-DN than those in higher NIS quartiles [33]. In a real-world clinical practice study from Belgium, patients had similar baseline characteristics and therapeutic response compared with the APOLLO study [34]. In another real-world case series, patisiran was used to successfully manage nine patients [20]. Patisiran also slowed down polyneuropathy progression post-liver transplant [35].

Similar to NIS, the mean difference of − 3.87 (± 9.2) in the KPS between baseline and the 48-month timepoint was significant. However, KPS was stable in 16 of 31 (51.6%) patients with the follow-up of 48 months, whilst two patients improved over the 4 years of observation.

Interestingly, the quality of life assessed using the Norfolk QoL-DN tool significantly improved at 12 and 24 months but returned to baseline levels at 48 months. ATTRv amyloidosis significantly impacts patients’ quality of life [36], but it is worth noting that follow-up of our population mostly occurred during the COVID-19 pandemic, which may have affected patients’ quality of life [37].

Autonomic dysfunction is an early, common and disabling feature of ATTRv amyloidosis that often manifests early in the course of the disease and has a substantial adverse impact on quality of life and survival. In our population, patisiran demonstrated to be able to halt autonomic impairment by improving CADT score by at least 1 point from baseline in 27.7% and stabilising within the range of ± 1 point in 44.8% of patients at 48 months (Supplementary Figure S6). Accordingly, in the APOLLO study, autonomic dysfunction was evaluated using the COMPASS-31 questionnaire and showed an improvement in 30% of patients in the orthostatic intolerance domain [38].

In the current study, no hitherto unknown safety signals were observed. The study drug-related extravasation observed in two patients was previously described in the Summary of Product Characteristics as an uncommon adverse event [39].

There are many strengths in our study. These include the real-world setting allowing for a realistic assessment of the effectiveness of patisiran in a relatively large study population and an unprecedentedly long follow-up of 4 years. On the other hand, the study has some limitations, such as the retrospective design with a potential for bias because of the possible inaccurate data records of patient notes and a small number of items analysed.

In conclusion, our data show that patisiran largely maintained the disease status in Italian patients with ATTRv amyloidosis.

### Supplementary Information

Below is the link to the electronic supplementary material.

Supplementary file1 (DOCX 7045 KB)

## Data Availability

The datasets generated during and/or analysed during the current study are available from the corresponding author upon reasonable request*.*
