# Correction to: Long-term treatment of hereditary transthyretin amyloidosis with patisiran: multicentre, real-world experience in Italy

**DOI:** 10.1007/s10072-024-07717-z

**Published:** 2024-08-02

**Authors:** Luca Gentile, Anna Mazzeo, Chiara Briani, Silvia Casagrande, Marcella De Luca, Gian Maria Fabrizi, Christian Gagliardi, Chiara Gemelli, Francesca Forcina, Marina Grandis, Valeria Guglielmino, Giacomo Iabichella, Luca Leonardi, Alessandro Lozza, Fiore Manganelli, Roberta Mussinelli, Filomena My, Giuseppe Occhipinti, Silvia Fenu, Massimo Russo, Angela Romano, Alessandro Salvalaggio, Matteo Tagliapietra, Stefano Tozza, Giovanni Palladini, Laura Obici, Marco Luigetti

**Affiliations:** 1 Unit of Neurology and Neuromuscular Diseases, Department of Clinical and Experimental Medicine, University of Messina, Messina, Italy; 2 Department of Neurosciences, Neurology Unit, University of Padova, Padua, Italy; 3 Department of Neurosciences, Psychology, Drug Research and Child Health (NEUROFARBA), University of Florence, Florence, Italy; 4 Department of Neurological Sciences, Biomedicine and Movement Sciences, University of Verona, Verona, Italy; 5 Cardiology Unit, IRCCS Azienda Ospedaliero-Universitaria of Bologna, Bologna, Italy; 6 IRCCS Policlinico San Martino Hospital, Genoa, Italy; 7 Department of Neuroscience, Mental Health and Sensory Organs (NESMOS), Sapienza University of Rome, Rome, Italy; 8 Dipartimento Di Neuroscienze, Riabilitazione, Oftalmologia, Genetica e Scienze Materno-Infantili, Università Di Genova, Genoa, Italy; 9 Dipartimento Di Neuroscienze, Università Cattolica del Sacro Cuore, Rome, Italy; 10 Amyloidosis Research and Treatment Centre, IRCCS Fondazione Policlinico San Matteo, Pavia, Italy; 11 Department of Neuroscience, Reproductive and Odontostomatological Science, University of Naples “Federico II”, Naples, Italy; 12 Department of Neurology, “Vito Fazzi” Hospital, Lecce, Italy; 13 S.C. Malattie Neurologiche Rare, Dipartimento di Neuroscienze Cliniche, Fondazione IRCCS Istituto Neurologico Carlo Besta, Milan, Italy; 14 Dipartimento Di Neuroscienze, Organi Di Senso e Torace, Fondazione Policlinico Universitario Agostino Gemelli IRCCS, Rome, Italy; 15 Department of Molecular Medicine, University of Pavia, Pavia, Italy

**Correction to: Neurological Sciences (2024)**

10.1007/s10072-024-07494-9

The article “Long‑term treatment of hereditary transthyretin amyloidosis with patisiran: multicentre, real‑world experience in Italy”, written by Luca Gentile, Anna Mazzeo, Chiara Briani, Silvia Casagrande, Marcella De Luca1, Gian Maria Fabrizi, Christian Gagliardi, Chiara Gemelli, Francesca Forcina, Marina Grandis, Valeria Guglielmino, Giacomo Iabichella, Luca Leonardi, Alessandro Lozza, Fiore Manganelli, Roberta Mussinelli, Filomena My, Giuseppe Occhipinti, Silvia Fenu, Massimo Russo, Angela Romano, Alessandro Salvalaggio, Matteo Tagliapietra, Stefano Tozza, Giovanni Palladini, Laura Obici and Marco Luigetti, was originally published electronically on the publisher’s internet portal on 16 April 2024 without open access. With the author(s)’ decision to opt for Open Choice the copyright of the article changed on 27 July 2024 to © The Author(s) 2024 and the article is forthwith distributed under a Creative Commons Attribution 4.0 International License, which permits use, sharing, adaptation, distribution and reproduction in any medium or format, as long as you give appropriate credit to the original author(s) and the source, provide a link to the Creative Commons licence, and indicate if changes were made. The images or other third party material in this article are included in the article’s Creative Commons licence, unless indicated otherwise in a credit line to the material. If material is not included in the article’s Creative Commons licence and your intended use is not permitted by statutory regulation or exceeds the permitted use, you will need to obtain permission directly from the copyright holder. To view a copy of this licence, visit http://creativecommons.org/licenses/by/4.0.

The original article has been corrected.

