# Supplementary material for: Long-term treatment of hereditary transthyretin amyloidosis with patisiran: multicentre, real-world experience in Italy
Source: Neurol Sci. 2024 Apr 16;45(9):4563–71. doi: 10.1007/s10072-024-07494-9 (PMC11306272; doi:10.1007/s10072-024-07494-9)
Supplement: Supplementary file 1 — Supplementary file1 (DOCX 7045 KB) [file 10072_2024_7494_MOESM1_ESM.docx]

**SUPPLEMENTARY MATERIALS**

***Neurological Sciences***

**Long-term treatment of hereditary transthyretin amyloidosis with patisiran: multicentre, real-world experience in Italy**

**Supplementary Table S1**. Participating centres.

| **Centre** | **Number of patients** | **Age**  **Median (IQR)**  **[years]** | **Gender** |
| --- | --- | --- | --- |
| C2 - Centro di Riferimento Regione Toscana per la diagnosi e cura dell’Amiloidosi, Firenze | 2 (5.0%) | 70 (67,73) | 2 M – 0 F |
| C3 - Fondazione Policlinico Universitario A. Gemelli IRCCS, UOC Neurologia, Roma | 4 (10%) | 70 (69,78) | 4 M – 0 F |
| C4 - IRCCS Ospedale Policlinico San Martino, Genova | 3 (7.5%) | 73 (72,78) | 2 M – 1 F |
| C5 - Neurologia Lecce | 2 (5.0%) | 74 (71,78) | 2 M – 0 F |
| C6 - UOC di Neurologia e Malattie Neuromuscolari, AOU G. Martino, Messina | 10 (25%) | 68 (52,73) | 8 M – 2 F |
| C7 - Milano | 2 (5.0%) | 68 (63,73) | 1 M – 1 F |
| C9 - Padova | 4 (10.0%) | 73 (66,75) | 2 M – 2 F |
| C8 - Napoli, Federico II | 1 (2.5%) | 68 (68,68) | 1 M – 0 F |
| C10 - IRCCS Fondazione Policlinico San Matteo, Pavia | 5 (12.5%) | 57 (57, 66) | 3 M – 2 F |
| C11 - Sant'Andrea-UOC Neurologia, Roma | 4 (10.0%) | 70 (69,72) | 3 M – 1 F |
| C12 - AOUI Verona | 3 (7.5%) | 79 (76,82) | 2 M – 1 F |

IQR, interquartile range

**Supplementary Table S2.** Baseline characteristics of patients included in this study.

| **Patient** | **Mutation** | | **Age** | **Time from diagnosis to patisiran (months)** | **Sex** | **Cardiomyopathy (yes/no)** | **FAP stage** | **PND** | **NIS** | **CADT** | **KPS** | **Norfolk QoL -DN** |
| --- | --- | --- | --- | --- | --- | --- | --- | --- | --- | --- | --- | --- |
| 1 | F64I | p.Phe84Ile | 64 | 4 | M | Yes | FAP1 | 1 | 45 | 9 | 60 | NA |
| 2 | V30M | p.Val50Met | 76 | 22 | M | Yes | FAP2 | 3a | 70 | 20 | 60 | NA |
| 3 | V30M | p.Val50Met | 70 | 96 | M | Yes | FAP1 | 2 | 77 | 10 | 60 | 70 |
| 4 | F64L | p.Phe84Leu | 69 | 72 | M | Yes | FAP2 | 3a | 86 | NA | 80 | 91 |
| 5 | F64L | p.Phe84Leu | 80 | 108 | M | Yes | FAP1 | 2 | 84 | 10 | 70 | 73 |
| 6 | F64L | p.Phe84Leu | 69 | 48 | M | Yes | FAP2 | 3a | 111 | 10 | 50 | NA |
| 7 | V30M | p.Val50Met | 73 | 48 | M | Yes | FAP1 | 2 | 25 | 19 | 80 | 31 |
| 8 | V30M | p.Val50Met | 72 | 60 | M | Yes | FAP2 | 3b | 88.5 | 7 | 60 | 74 |
| 9 | F64L | p.Phe84Leu | 82 | 48 | F | Yes | FAP1 | 1 | 13 | 15 | 95 | 10 |
| 10 | F64L | p.Phe84Leu | 68 | 57 | M | No | FAP1 | 2 | 80 | 16 | 60 | 50 |
| 11 | F64L | p.Phe84Leu | 81 | 96 | M | No | FAP1 | 2 | 80 | 11 | 60 | 68 |
| 12 | F64L | p.Phe84Leu | 78 | 12 | M | No | FAP2 | 3b | 94 | 13 | 60 | 64 |
| 13 | F64L | p.Phe84Leu | 74 | 12 | M | No | FAP2 | 3a | 74 | 13 | 60 | 50 |
| 14 | E89Q | p.Glu109Gln | 53 | 6 | M | No | FAP1 | 1 | 36 | 13 | 70 | 58 |
| 15 | F64L | p.Phe84Leu | 69 | 6 | M | Yes | FAP2 | 3b | 72 | 10 | 50 | 78 |
| 16 | F64L | p.Phe84Leu | 68 | 48 | M | No | FAP1 | 2 | 63 | 16 | 70 | 48 |
| 17 | V122I | p.Val142Ile | 70 | 12 | M | Yes | FAP2 | 3b | 56 | 12 | 50 | 60 |
| 18 | F64L | p.Phe84Leu | 52 | 72 | M | No | FAP1 | 1 | 36 | 14 | 80 | 54 |
| 19 | E89Q | p.Glu109Gln | 48 | 24 | F | Yes | FAP2 | 3a | 97 | 7 | 40 | 100 |
| 20 | V30M | p.Val50Met | 75 | 36 | M | Yes | FAP1 | 2 | 103 | 13 | 60 | 94 |
| 21 | E89Q | p.Glu109Gln | 50 | 12 | F | No | FAP2 | 3a | 105 | 8 | 40 | 108 |
| 22 | F64L | p.Phe84Leu | 78 | 48 | M | Yes | FAP1 | 2 | 66 | 18 | 90 | 57 |
| 23 | E89K | p.Glu109Lys | 58 | 72 | F | Yes | FAP1 | 2 | 53 | 15 | 90 | 45 |
| 24 | V30M | p.Val50Met | 68 | 8 | M | Yes | FAP2 | 3b | 100 | 17 | 60 | 111 |
| 25 | E89Q | p.Glu109Gln | 50 | 28 | M | Yes | FAP1 | 1 | 11 | 16 | 90 | 5 |
| 26 | S77Y | p.Ser77Tyr | 74 | 72 | F | Yes | FAP1 | 1 | 27 | 17 | 80 | 8 |
| 27 | V30M | p.Val50Met | 72 | 46 | M | No | FAP2 | 3b | 118 | 9 | 70 | 49 |
| 28 | F64L | p.Phe84Leu | 79 | 32 | F | Yes | FAP2 | 3a | 47 | 11 | 70 | 27 |
| 29 | T49A | p.Thr69Ala | 49 | 72 | F | Yes | FAP1 | 2 | 46 | 17 | 90 | 65 |
| 30 | F64L | p.Phe84Leu | 66 | 36 | M | No | FAP1 | 2 | 65 | 12 | 80 | 48 |
| 31 | F64L | p.Phe84Leu | 57 | 48 | M | No | FAP1 | 2 | 80 | 12 | 80 | 90 |
| 32 | E89Q | p.Glu109Gln | 57 | 60 | M | Yes | FAP1 | 2 | 60 | 16 | 80 | 85 |
| 33 | V30M | p.Val50Met | 76 | 120 | F | Yes | FAP2 | 3b | 130 | 15 | 50 | 83 |
| 34 | V30M | p.Val50Met | 71 | 36 | M | Yes | FAP1 | 2 | 62.5 | 10 | 60 | 54 |
| 35 | F64L | p.Phe84Leu | 74 | 68 | M | Yes | FAP1 | 2 | 82 | 11 | 70 | 65 |
| 36 | E89Q | p.Glu109Gln | 67 | 48 | F | Yes | FAP1 | 2 | 79 | 16 | 60 | 69 |
| 37 | V30M | p.Val50Met | 70 | 60 | M | Yes | FAP2 | 3b | 83 | 13 | 50 | 67 |
| 38 | Y78F | p.Thyr98Phe | 73 | 36 | M | No | FAP1 | 2 | 76 | 14 | 70 | 68 |
| 39 | F64L | p.Phe84Leu | 85 | 132 | F | Yes | FAP2 | 3b | 103 | 14 | 60 | 70 |
| 40 | Y78F | p.Thyr98Phe | 79 | 48 | M | Yes | FAP2 | 3a | 73 | 16 | 70 | 55 |

Legend: CADT, Composite Autonomic Dysfunction Test; FAP, familial amyloidosis polyneuropathy; IVS, intraventricular septum; KPS, Karnofsky performance status; NIS, neuropathy impairment score; PND, polyneuropathy disability; SD, standard deviation; QoL-DN – quality of life – diabetic neuropathy.

**Supplementary Table S3**. Polyneuropathy disability (PND) class and familial amyloid polyneuropathy (FAP) stage evolution in individual patients. The patient who improved regarding the PND score is shown in bold.


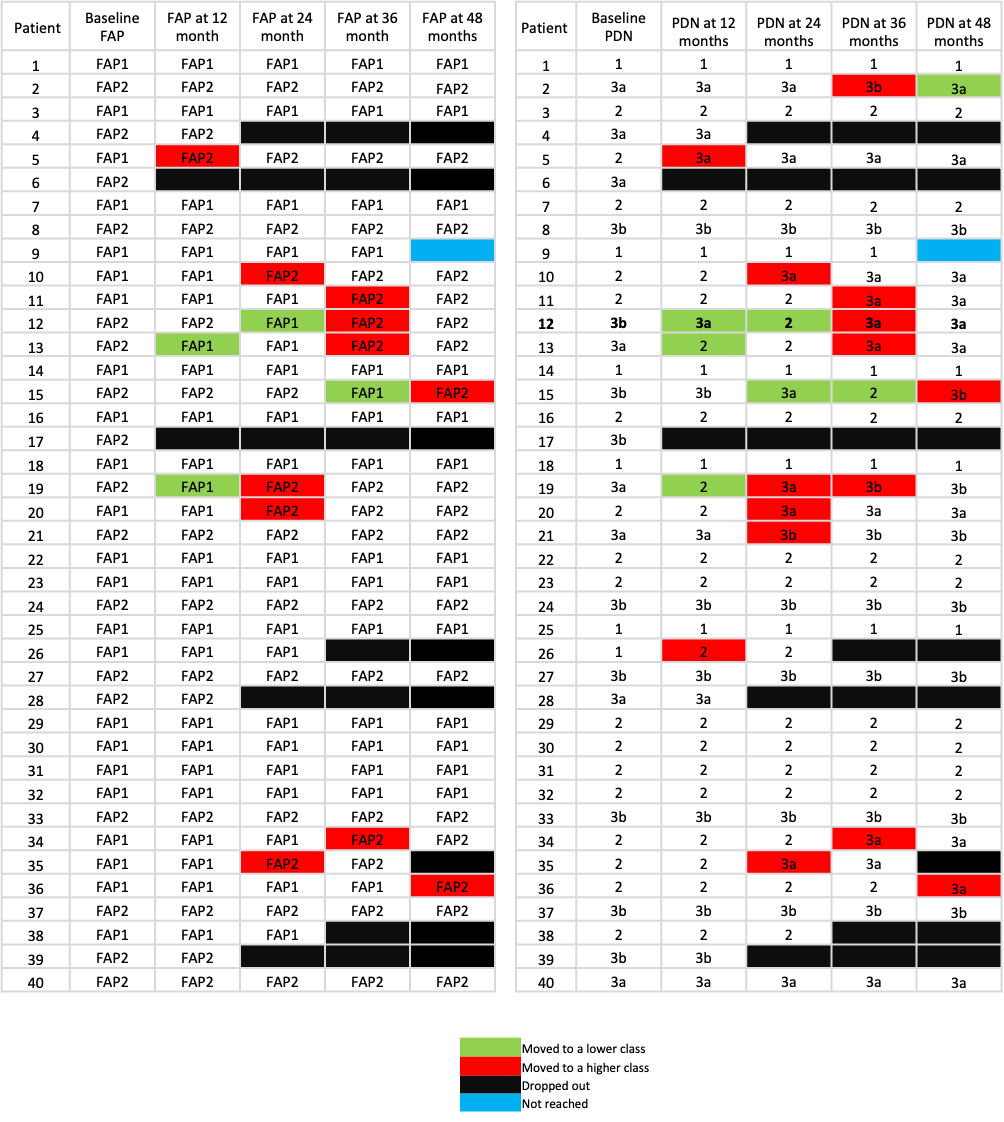


**Supplementary Table S4**. Summary of adverse events.

| **Adverse event** | **Number of patients (%)** | **Serious adverse event** | **Intensity** | **Relation to study drug** | **Action on drug administration** | **Outcome** |
| --- | --- | --- | --- | --- | --- | --- |
| Sepsis | 1 (2.3) | Yes | Severe | Unlikely | Suspended | Fatal |
| Stroke and pneumonia | 1 (2.3) | Yes | Severe | Unlikely | Suspended | Fatal |
| Sudden death | 1 (2.3) | Yes | Severe | Unlikely | Suspended | Fatal |
| Severe diarrhoea leading to hypovolemic shock | 1 (2.3) | Yes | Severe | Unrelated | None | Fatal |
| Stroke | 1 (2.3)* | Yes | Severe | Unrelated | None | Resolved |
| Recurrent vomiting (eight episodes) | 1 (2.3) | Yes | Moderate | Unrelated | Suspended on 1/8 occasions | Resolved |
| Drug extravasation reaction | 1 (2.3) | No | Moderate | Certain | None | Resolved |
| Panniculitis due to extravasation | 1 (2.3) | No |  | Certain | None | Resolved |
| Mild nausea/vomiting the day after infusion | 1 (2.3) | No | Mild |  | None | Resolved |
| Hyperglycaemia due to steroid pre-treatment | 1 (2.3) | No |  | Unrelated | None | Resolved |

*Patient on ASA prophylaxis

**Supplementary Figure S1**. The proportion on patients in PND classes 1 to 4 over the follow-up of 40 months.

**Supplementary Figure S2**. The evolution from baseline of NIS score. A. Evolution in individual patients with baseline and 48-month assessment (n=31). B. Percentages of patients with improved, stable, or worsened neuropathy impairment score (NIS) at 48 months.

A


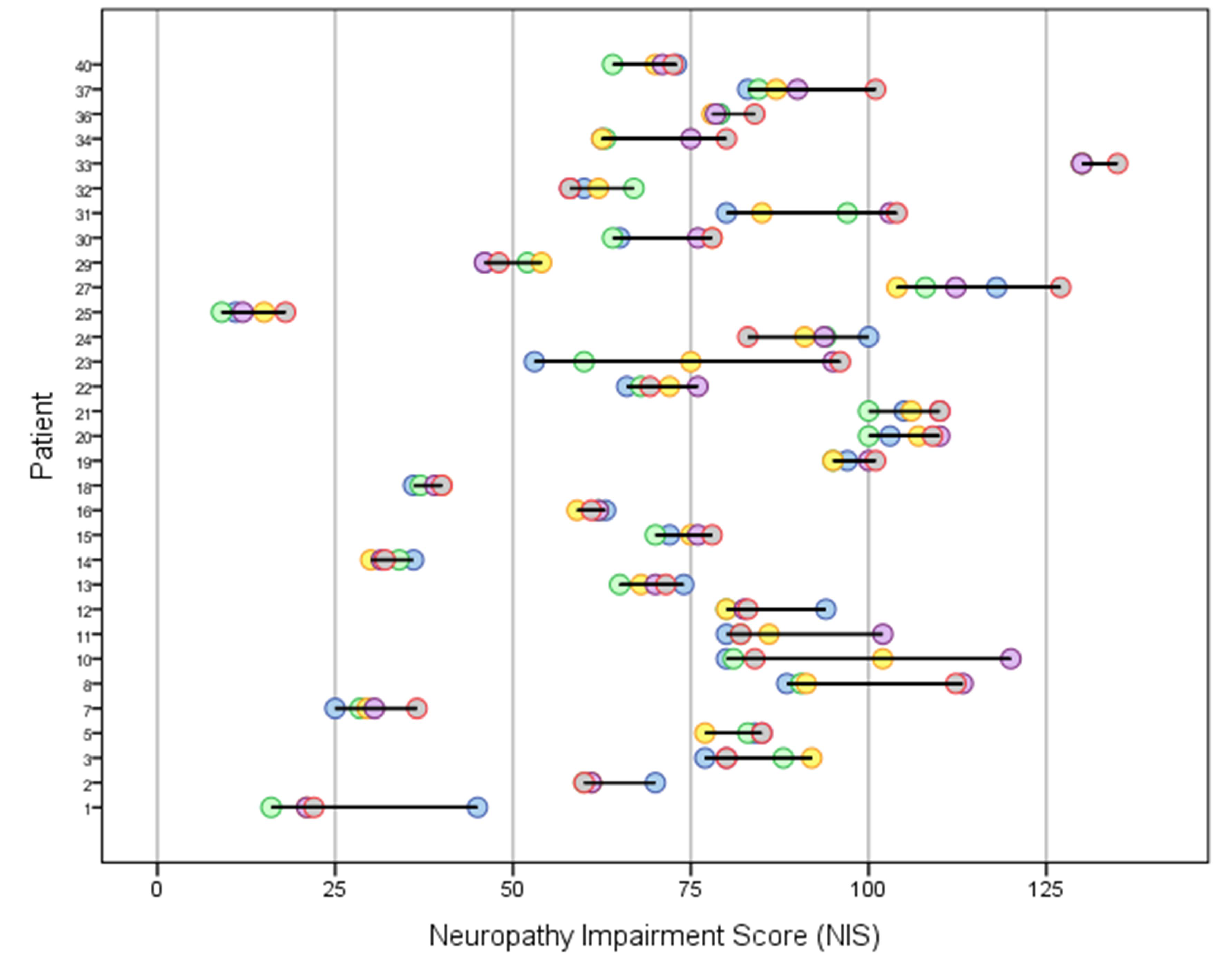


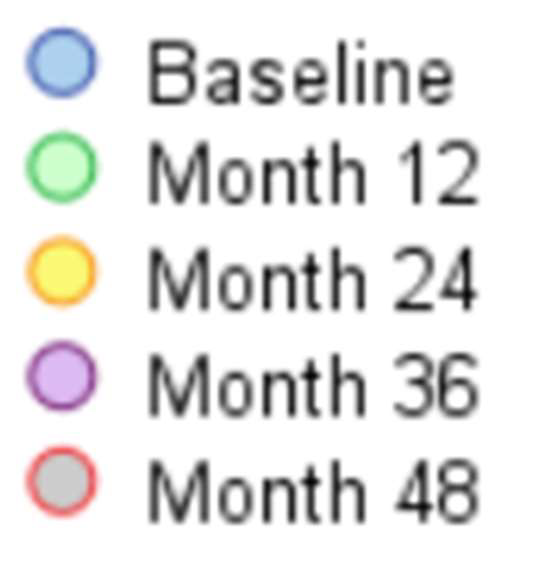


B

**Supplementary Figure S3**. The evolution from baseline of mBMI in patients with baseline and 48-month assessment (n=24).


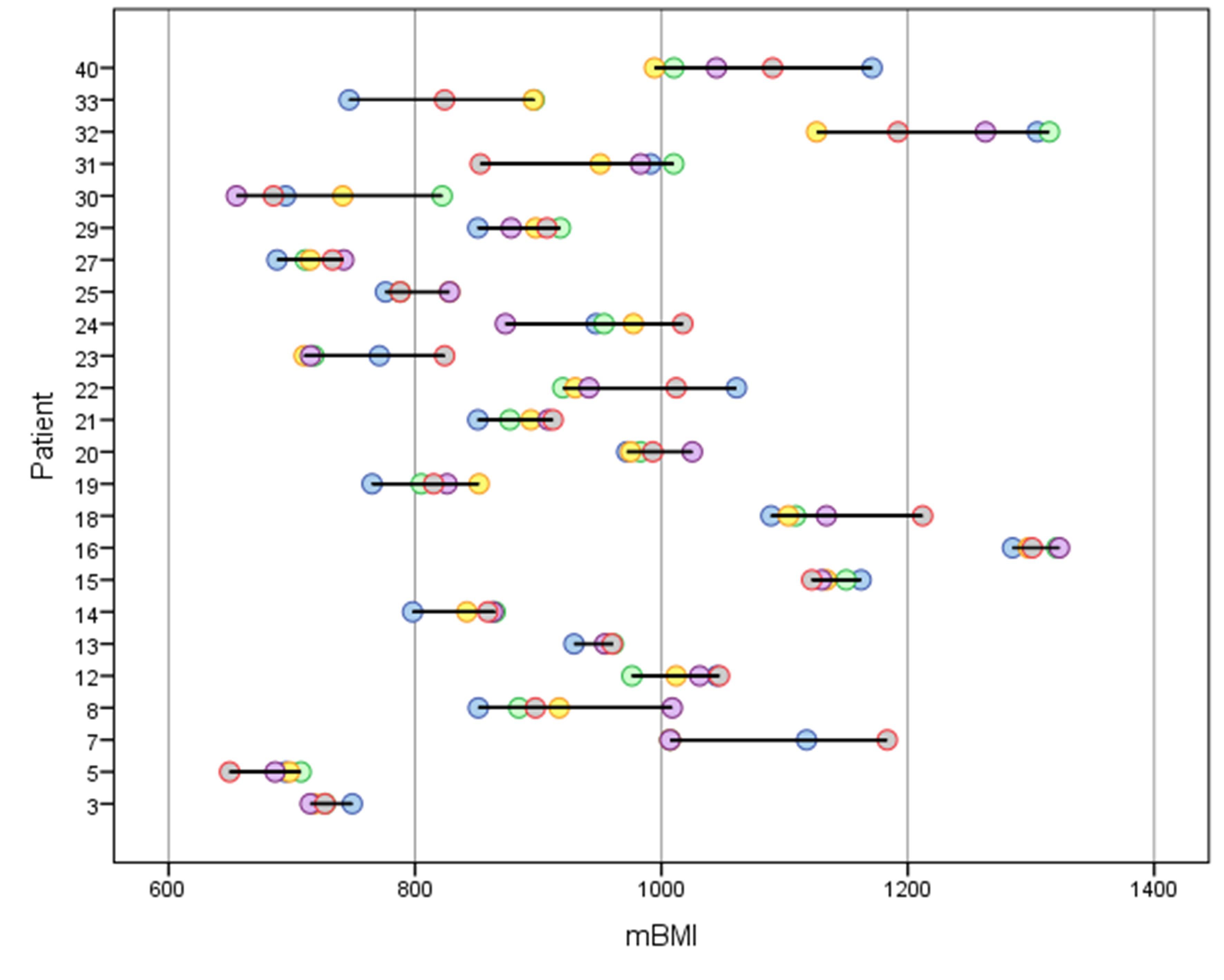


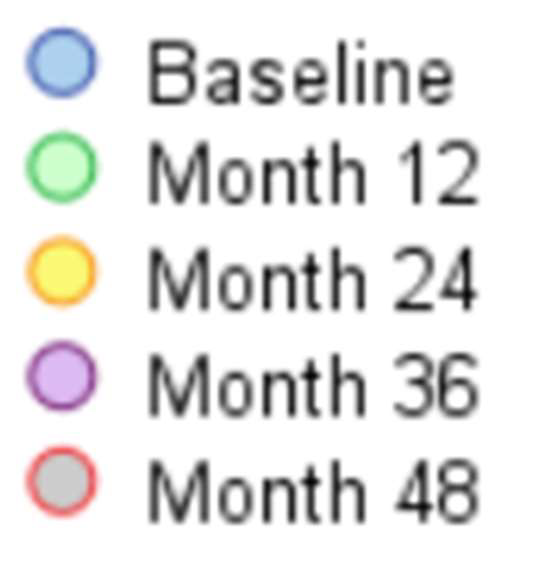


**Supplementary Figure S4**. The evolution from baseline of KPS in patients and baseline and 48-month assessment (n=31). In patients for whom only one red dot is visible, the score remained unchanged.


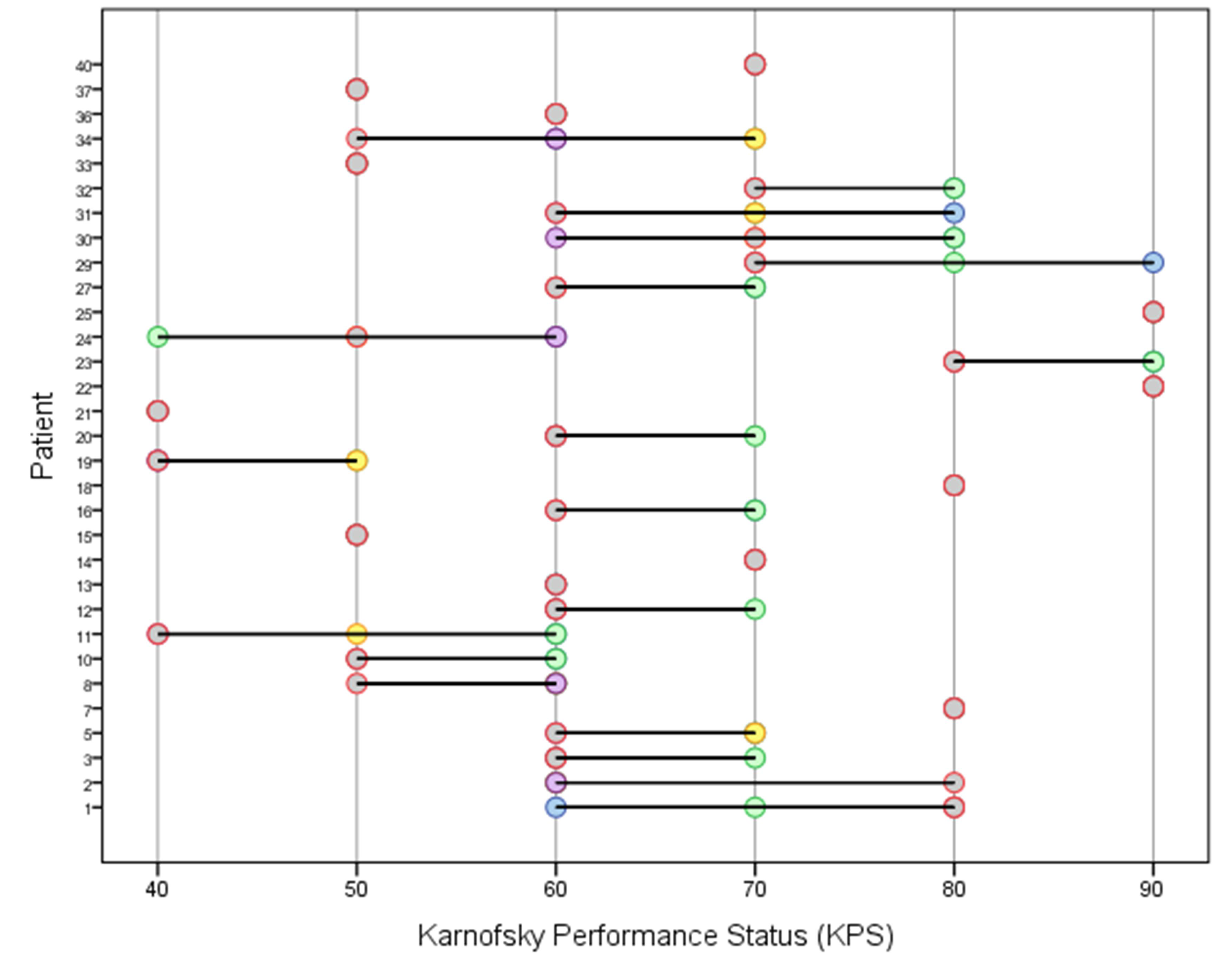


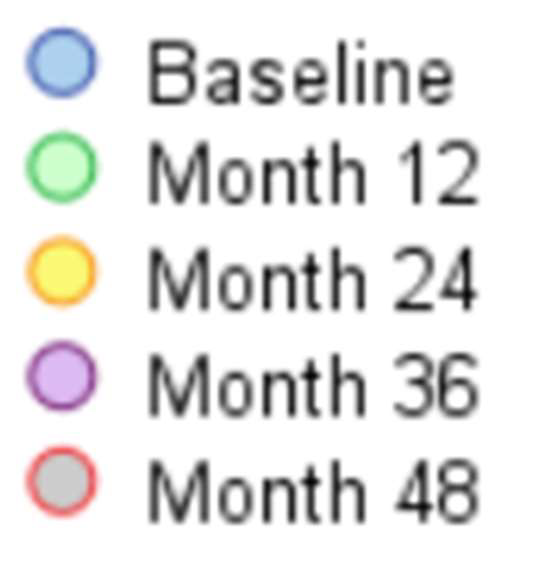


**Supplementary Figure S5**. The evolution from baseline of Norfolk QoL-DN score in all patients with a follow-up of 48 months (n=27).


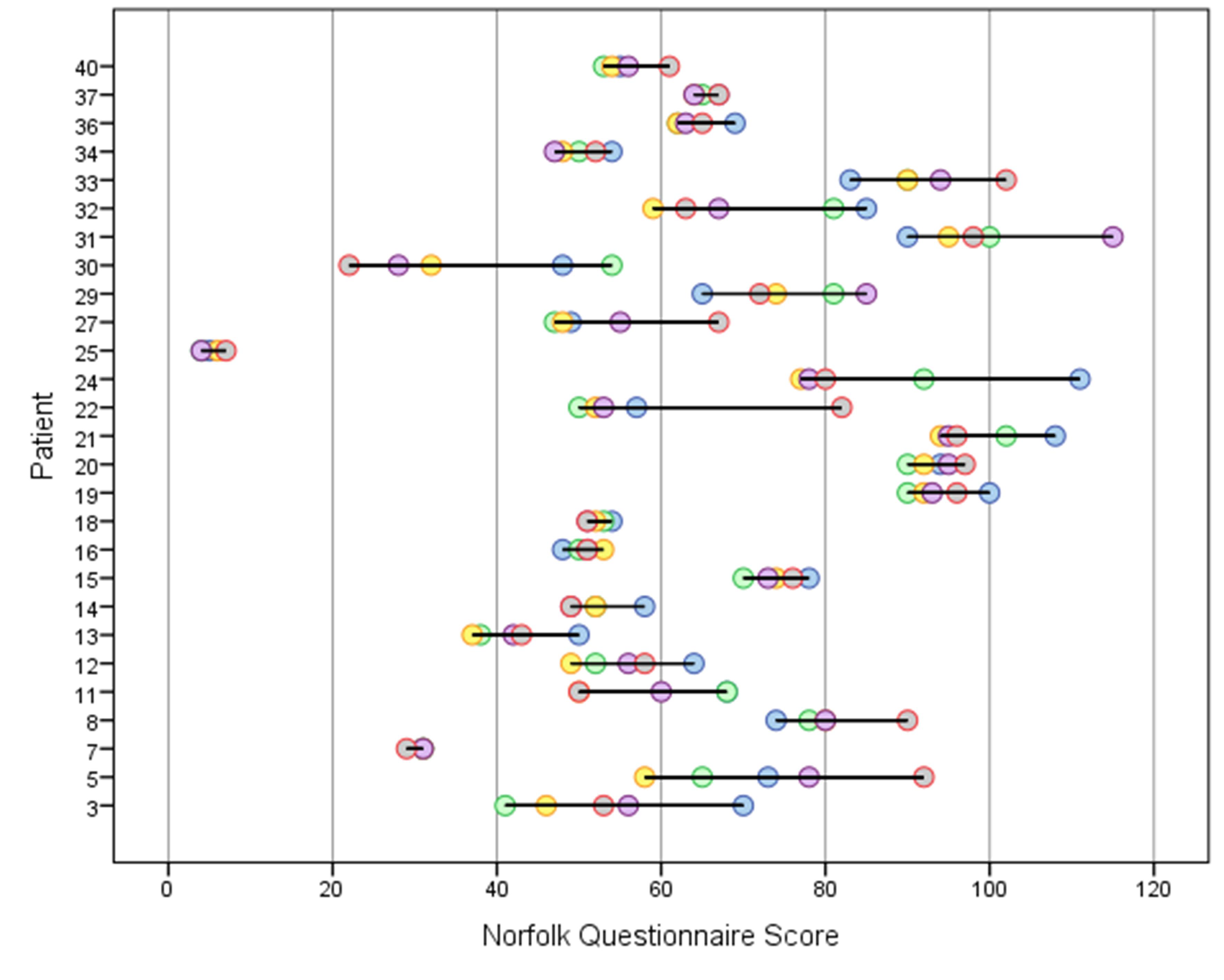


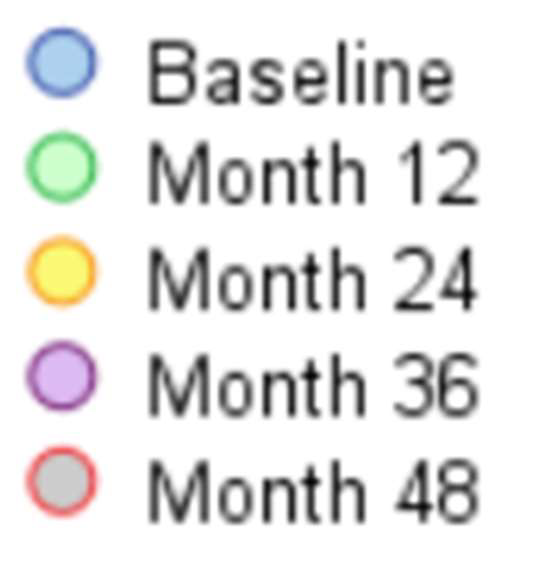


**Supplementary Figure S6**. The evolution from baseline of CADT. A. Evolution in all individual patients with baseline and follow-up data at 48 months (n=29). B. Percentages of patients with improved, stable, or worsened CADT at 48 months.

A

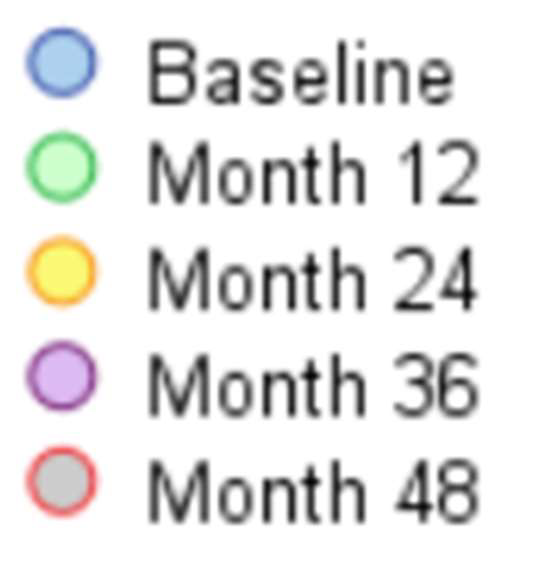


B
